# Mr. Gray and Mr. Ellis, on Certain Diseases of the African Slaves

**Published:** 1816-05

**Authors:** Henry Ellis


### *Mr.* GRAY *and Mr.* ELLIS, *on certain Diseases of the African Slaves.*

---

### *Extract of a Letter from Mr.* GRAY, *Surgeon R. N. to Mr.* JOHNSON.

Brockhurst, March 6, 1816.

" DEAR SIR,

" AGREEABLY to your request, I forward you a few remarks, which I made during my stay on the African station last year.

" We made the coast about the middle of February, with the thermometer at 76; and our men continued healthy during the remainder of February and March, at which time the Harmattan winds were prevalent. In the beginning of April, after leaving the river of Old Callabar, where we released four hundred slaves, dysentery made its appearance with great violence. Our men had been forced to undergo much fatigue in the heat of the sun, with the mercury ranging from 100 to 103 in the day; while a copious precipitation of heavy dews took place at night, causing a rawness and comparative coldness of the atmosphere. Vast tracts of country, on both sides of the river, were covered with mud, intermixed with vegetable and animal remains, from which an effluvium constantly issued, that was intolerably offensive to the smell. On this account, it needs not excite surprize, that with dysentery there was combined a bilious remittent and intermittent fever; particularly in the last stage of the disease, when the respiration became laborious; the tongue covered with a dark mucus; the uvula invariably elongated, and of a pale yellow colour, especially in the morning. Before death, this yellow colour was very visible all over the fauces and pharynx, extending in right lines along the œsophagus to the stomach itself, with an entire destruction of the villous membrane, of which I had numerous proofs, while examining the bodies of the slaves who died on board with symptoms precisely similar to those observed in the Europeans. The progress of emaciation was astonishingly rapid in this disease, both among the blacks and whites. All the Europeans complained of excruciating pain in the umbilical region ; and that the same was the case among the slaves was evinced by their lying on the deck with the hands constantly pressed on the abdomen, or sitting couched like so many monkies in the most deplorable and disgusting state, voiding their fæces involuntarily, and exhibiting a scene of human wretchedness which might affect the callous heart of even a trafficker in human flesh!

"After some dear-bought experience, and for reasons which will presently appear, I pursued the following plan among my negro patients: viz. Half a grain of the oxymurias hydrargyri was given night and morning, exhibiting frequent emollient enemas to palliate the tenesmus, which was a most distressing symptom. The good effects of this treatment were evident so soon as the system became saturated with the medicine. The countenance cleared up, and assumed a degree of serenity ; and the poor creatures endeavoured to manifest their gratitude for delivery from agony, by pressing my hand to their hearts.

"My success by this mode of treatment exceeded my expectations, and I am inclined to attribute it, in a great measure, to the peculiar effects of the oxymuriate as a *vermifuge;* for almost invariably, on the third or fourth day from the commencement of its exhibition, astonishing quantities of a small species of ascarides were voided by stool, in balls resembling scybala.

"On examining the bodies after death, I generally found the mesentery much wasted, with quantities of ascarides about the pyloric orifice of the stomach and arch of the colon. I lost very few Europeans, which prevented me having opportunities of dissection in them ; but as the symptoms were the same in both classes of patients, it is fair to presume that the *post mortem* appearances would have been the same. I enclose you the remarks of a brother officer on the same subject, who had been a considerable time on the coast, with ample opportunities for observation."

*Extract of a Letter from Mr.* HENRY ELLIS, *Surgeon R. N. to Mr.* GRAY.

May 10, 1815.

"AMONG the slaves which we lately carried into Sierra Leone, worms, scurvy, and dysentery, were the principal complaints that required medical attention. I have no hesitation in stating my belief that dysentery was generally symptomatic of worms, or scurvy—particularly of worms. Not being able to gain any oral information from these unfortunate creatures, and finding those whom I first visited lying on the deck, reduced to an extreme degree of emaciation, and passing their stools involuntarily, I naturally concluded that the complaint was dysentery in its last stage. To these I first exhibited tinct. opii in a glass of Port wine, and found it gave a temporary relief: I also gave pulv. rhei combined with opium, which seemed serviceable. But on a more intimate acquaintance with the disease, I had reason to suspect that the primary cause was worms; and therefore I gave the submuriate of quicksilver every second or third day, with the intention of destroying them, exhibiting the sedatives in the intervals, to allay the intestinal irritation. The oxymuriate of quicksilver, which, on other and subsequent occasions, I found so powerful a vermifuge, I was afraid to venture on here, lest the alimentary canal, denuded of its mucous membrane, should not be in a state to bear it. From the mucilagino-saccharine quality of the vegetables on which these Africans exclusively feed, an extensive nidus must exist in the intestinal tube, and every circumstance seems favourable to the generation and propagation of worms. But when these unhappy wretches enter on their captivity, and their diet is entirely changed, the mucus and mucilage, before so copious in the primæ viæ, become suddenly deficient, and then the worms make dreadful ravages on the intestines themselves. Of this I had the most convincing proofs in the numerous dissections which I made among the slaves who died. In almost every case, the inner coat of the alimentary canal was completely abraded, and the points of the vessels seen filled with blood. Wherever the worms were lodged, there the intestines were so thin that, in very many cases, they could be distinctly seen through the remaining coats.

"A return to their usual diet (plantains and yams), even in apparently desperate cases, soon produced the most surprising good effects; of which we had a striking instance lately at Cape Coast, where, in one week of vegetable diet, the slaves were restored to pristine health, from a state of great vermino-dysenteric irritation and debility.

"When one of these poor afflicted creatures came to me with his hand pressed hard on the umbilicus, leaning forward and writhing with pain, the pulse full, the skin hot, tongue foul, and stools frequent, I had no doubt respecting the nature of the disease, as resulting from the irritation of worms in the intestines. My practice was, to exhibit, in the morning, a quarter of a grain of the oxymuriate of quicksilver, in the form of a pill; and, in the evening, double that quantity of the same medicine. On the following morning, a brisk cathartic was given, which seldom failed to bring off great quantities of worms, with marked relief to the patient. In this way, I saved the lives of numbers, when I found them in an early stage of the disease, viz. when the stools appeared liquid and gritty, as though they contained a mixture of brownish earth. But in an advanced stage of this disease, the stools became watery, greenish, and mixed with white coagulated particles. Here the whole track of the primæ viæ appeared in a state of inflammation or ulceration; for, on looking into the mouth, the posterior fauces and pharynx appeared red, swelled, and sometimes ulcerated; the whole exhibiting the appearance which a part severely scalded would assume on the third or fourth day. In addition to these symptoms, there was, in this advanced stage, a great flow of viscid phlegm from the mouth. In these periods of the disease, the oxymuriate could not be borne, and calomel was my only refuge; but, as may be expected, I often failed to effect a cure.

"The dysentery consequent on scurvy, among the negro slaves, is usually rapid in its progress, and certain in its fatal termination. Here *palliation* by the citric acid was all I could aim at. One bottle of lime-juice would *prevent* more cases of scurvy than forty would *cure*.

"The condition in which we frequently found these unhappy wretches, was often deplorable and degrading to humanity! On taking possession of a vessel, most of the male negroes were observed to be in irons between decks. In the mornings, indeed, the women and children were dragged upon deck from their human, or rather inhuman stew-holes. These poor creatures would huddle themselves together, like a parcel of monkies or frightened fowls, and there remain squatted in the same position, till ordered back again to their dismal abode! Among such desponding wretches, scurvy made rapid strides; indeed, we frequently found them so over-run with this loathsome disease, that the whole of their teeth might be picked from the gums without causing the smallest degree of pain.

"HENRY ELLIS."

---

Such are the scenes which, at this enlightened æra of Christianity, are still presented on the ill-fated shores of Africa! such the trade, which our *Christian* allies still drive in human flesh — in human blood! It is one consolation, that the wooden bulwarks of England, which too long protected this iniquitous commerce, are now employed in severing the chains of our African fellow-creatures, and wresting from the claws of European harpies the innocent victims of their insatiate oppressors.

EDITORS.

